# Dual Antiplatelet Therapy (DAPT) versus No Antiplatelet Therapy and Incidence of Major Bleeding in Patients on Venoarterial Extracorporeal Membrane Oxygenation

**DOI:** 10.1371/journal.pone.0159973

**Published:** 2016-07-28

**Authors:** Dawid L. Staudacher, Paul M. Biever, Christoph Benk, Ingo Ahrens, Christoph Bode, Tobias Wengenmayer

**Affiliations:** 1 Heart Center Freiburg University, Department of Cardiology and Angiology I, Freiburg, Germany; 2 Heart Center Freiburg University, Department of Cardiovascular Surgery, Freiburg, Germany; Medizinische Hochschule Hannover, GERMANY

## Abstract

**Aims:**

Bleeding is a frequent complication in patients on venoarterial extracorporeal membrane oxygenation (VA-ECMO). An indication for dual antiplatelet therapy due to coronary stent implantation is present in a considerable number of these patients. The objective of this retrospective study was to evaluate if dual antiplatelet therapy (DAPT) significantly increases the high intrinsic bleeding risk in patients on VA-ECMO.

**Methods and Results:**

A total of 93 patients were treated with VA-ECMO between October 2010 and October 2013. Average time on VA-ECMO was 58.9 ± 1.7 hours. Dual antiplatelet therapy was given to 51.6% of all patients. Any bleeding was recorded in 60.2% of all patients. There was no difference in bleeding incidence in patients on DAPT when compared to those without any antiplatelet therapy including any bleeding (66.7% vs. 57.1%, p = 0.35), BARC3 bleeding (43.8% vs. 33.3%, p = 0.31) or pulmonary bleeding (16.7% vs. 19.0%, p = 0.77). This holds true after adjustment for confounders. Rate of transfusion of red blood cells were similar in patients with or without DAPT (35.4% vs. 28.6%, p = 0.488).

**Conclusions:**

Bleeding on VA-ECMO is frequent. This registry recorded no statistical difference in bleeding in patients on dual antiplatelet therapy when compared to no antiplatelet therapy. When indicated, DAPT should not be withheld from VA ECMO patients.

## Introduction

There are several indications for venoarterial extracorporeal membrane oxygenation (va-ECMO) [1–4]. In patients with cardiogenic shock or after cardiopulmonary resuscitation, current guidelines advocate the consideration of a coronary angiography [5, 6] and a subsequent percutaneous coronary intervention (PCI) when indicated [7]. Therefore, a substantial subset of va-ECMO patients will undergo PCI and will have an indication for dual antiplatelet therapy (DAPT). In addition, current va-ECMO guideline recommends a treatment with unfractionated heparin for prevention of arterial thromboembolism [8]. ECMO by itself can cause coagulopathies [9–12] and bleeding incidence on therapy is high [13–16]. According to registry data of patients without ECMO, bleeding incidence in patients on DAPT in combination with oral anticoagulation is significantly higher compared to either DAPT alone or sole oral anticoagulation [17–19]. Whether bleeding on va-ECMO therapy is significantly increased by addition of DAPT to unfractionated heparin is unclear.

## Methods

We report data of a single center registry of patients on VA-ECMO. All patients presented at the Heart Center Freiburg University between October 2010 and October 2013. Data derived from the registry was blinded to patient identity. Data analysis (without direct patient follow up after index hospitalization) was approved by the Ethics Committee University of Freiburg (EK-Freiburg 151/14). Data analysis was performed using either t-test, ANOVA or Chi^2^-test as applicable and a p-value of < 0.05 was considered statistically significant. Data is given as mean ± standard error of the mean, when not indicated otherwise.

### Patient selection

Within October 2010 and October 2013, a total of 93 patients underwent va-ECMO implantation at the Heart Center University of Freiburg. Indication for va-ECMO was driven by the decision of the responsible physicians being part of our ECMO response team. Cannulation for va-ECMO was performed in Seldinger technique without surgical cut down. ECMO removal was performed as previously reported [14]. Most patients were either on DAPT or no antiplatelet therapy. Only 3 out of 93 patients had a therapy with only a single antiplatelet drug (being acetylsalicylic acid). Those 3 patients were included in the analysis regarding all patient data but excluded when comparing patients with DAPT to those without any antiplatelet therapy.

### Bleeding

Bleeding incidence was evaluated by manual search of medical and patient records. Bleedings were categorized using the BARC [20] classification (brief: BARC0—no bleeding; BARC1 –minimal bleeding; BARC2 –bleeding that needs further diagnostic or therapeutic steps, BARC3 –Bleeding plus drop in hemoglobin; BARC4 –CABG related bleeding, BARC5 –fatal bleeding). Underreporting of minor bleedings (BARC1) can methodically not be excluded. A drop of hemoglobin > 3 mg/dl or any red blood cell transfusion was considered to be a major bleeding (BARC3).

### Anticoagulation and transfusion

Unfractionated heparin was given to all patients aiming at a partial thromboplastin time of at least 40–50 seconds during VA-ECMO therapy. In case of spontaneous lengthening of the partial thromboplastin time, repeated measurements were performed every 8 hours and heparin was started as soon as partial thromboplastin time fell down to 50 seconds. Plasmatic coagulation including fibrinogen and platelets were recorded at least daily. In all patients, INR was normalized, fibrinogen was kept above 100 mg/dl and platelet count above 50 thousand/μl. In case of bleeding, we aimed for fibrinogen levels of 150 mg/dl and a platelet count of 80 thousand/μl. During VA-ECMO therapy, we aimed for hemoglobin levels of > 8 g/dl. Antiplatelet drugs were given at recommended loading and maintenance doses [7].

## Results

A total of 93 patients (mean age 57.8 ± 1.7 years) underwent VA-ECMO implantation (patients’ characteristics given in Table 1). 48 patients received DAPT and 42 patients received no antiplatelet therapy. Indication for DAPT was implantation of a drug eluting stent in 77.1% of all patients followed by bare metal stent implantation (16.7%). Other indications were recent TAVI (2.1%), non-ST elevation myocardial infarction with unsuccessful bypass PCI (2.1%) and drug eluting balloon PCI (2.1%). Comparing patients receiving DAPT to those without any antiplatelet therapy, those on DAPT were significantly older (66.2 ± 0.2 vs. 48.2 ± 0.4 years, p < 0.001). Other characteristics like gender, known coronary or peripheral artery disease, pulmonary or kidney disease and SAPS2 score did not differ between the groups.

**Table 1:** Patients’ characteristics.

	All patients	DAPT	no DAPT	p
Number of patients	93 (100.0%)	48 (51.6%)	42 (45.2%)	
**Patient characteristics**				
Age [years]	57.8 ± 1.7	48.2 ± 0.4	66.2 ± 0.2	**< 0.001**
Male gender	68 (73.1%)	33 (68.8%)	32 (76.2%)	0.432
SAPS2 Score	49.2 ± 1.5	50.1 ± 2.9	49.1 ± 1.6	0.741
CPR duration [min]	41.0 ± 0.8	42.6 ± 1.4	39.3 ± 1.9	0.672
Known CAD	35 (37.6%)	18 (37.5%)	16 (38.1%)	0.954
PAD	8 (8.6%)	5 (10.4%)	3 (7.1%)	0.586
COPD	2 (2.2%)	1 (2.1%)	1 (2.4%)	0.904
Other pulmonary disease	6 (6.5%)	4 (8.3%)	2 (4.8%)	0.498
Liver disease	3 (3.2%)	1 (2.1%)	2 (4.8%)	0.480
Kidney disease	26 (28%)	16 (33.3%)	9 (21.4%)	0.208
Arterial hypertension	50 (53.8%)	28 (58.3%)	20 (47.6%)	0.309
Diabetes	32 (34.4%)	17 (35.4%)	15 (35.7%)	0.977
**Indication for ECMO**				
IHCA	36 (38.7%)	26 (54.2%)	10 (23.8%)	**0.003**
OHCA	32 (34.4%)	15 (31.3%)	17 (40.5%)	0.362
Cardiogenic shock	16 (17.2%)	7 (14.6%)	6 (14.3%)	0.968
Septic shock	5 (5.4%)	0 (0.0%)	5 (11.9%)	**0.014**
Other	4 (4.3%)	0 (0.0%)	4 (9.5%)	**0.029**
**Antiplatelet drugs**				
Aspirin	51 (54.8%)	48 (100.0%)	0 (0.0%)	**< 0.001**
Clopidogrel	15 (16.1%)	15 (31.3%)	0 (0.0%)	**< 0.001**
Ticagrelor	17 (18.3%)	17 (35.4%)	0 (0.0%)	**< 0.001**
Prasugrel	16 (17.2%)	16 (33.3%)	0 (0.0%)	**< 0.001**

Patients’ characteristics are given as number of patients (percentage of all patients in group) or as mean ± standard deviation where applicable; statistical analysis is performed for ‘DAPT’ and ‘no antiplatelet therapy’. CPR—cardiopulmonary resuscitation; CAD—coronary artery disease; PAD—peripheral arterial disease; COPD—chronic obstructive pulmonary disease; IHCA—in-hospital cardiac arrest; OHCA—out-of hospital cardiac arrest

Most common indications for VA-ECMO implantation were out-of-hospital or in-hospital cardiac arrest without return of spontaneous circulation or recurrent need for cardiopulmonary resuscitation (34.4% and 38.7%, respectively). Other indications for VA-ECMO were cardiogenic or septic shock (17.2% and 5.4%, respectively) refractory to conservative therapies including vasopressors, positive inotropic agents and intra-venous volume therapy. The DAPT group contained significantly more patients after IHCA (p = 0.003) and fewer patients with septic shock (p = 0.014, Table 1). Mean CPR duration did not differ between the groups (DAPT 42.56 ± 9.76 min; no DAPT 39.26 ± 12.48; p = 0.672).

**Fig 1:**
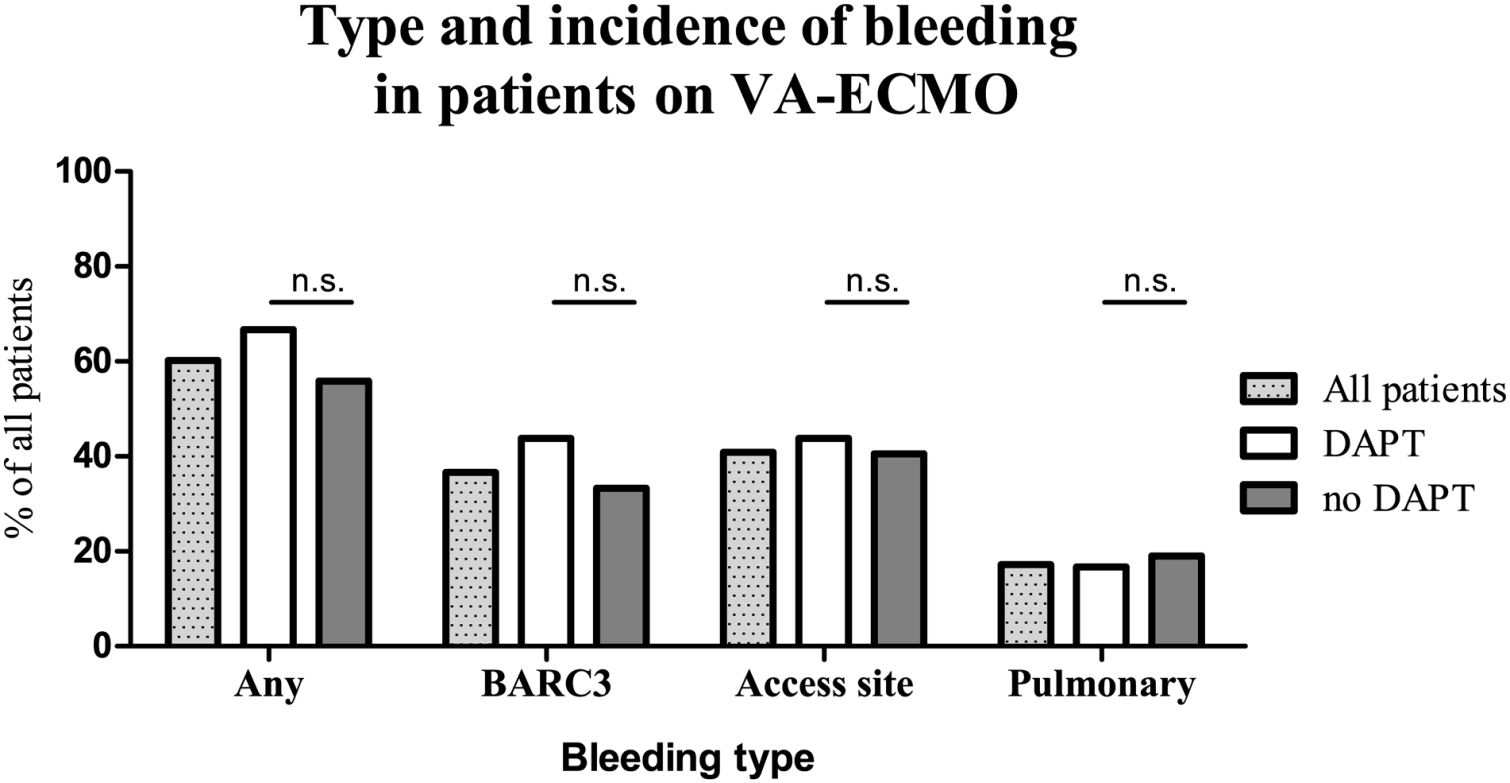
Bleeding incidence on venoarterial extracorporeal membrane oxygenation therapy, bleeding incidence during venoarterial extracorporeal membrane oxygenation therapy is given as percentage of patients with bleeding to total patients treated.

Average VA-ECMO therapy time was 55.6 ± 6.9 hours (median 37.1 hours) and did not differ significantly between the groups (DAPT 64.2 vs. no DAPT 41.9 hours, p = 0. 105). Aspirin or DAPT was given to slightly more than half of all patients (54.8% and 51.6%, respectively). DAPT was composed of aspirin plus either clopidogrel (31.3%), ticagrelor (35.4%) or prasugrel (33.3%). Any bleeding complications were recorded in a total of 60.2% of all patients while hemoglobin relevant bleedings (BARC3) were found in 36.6%. Any bleeding incidence was not statistically different in patients on DAPT when comparing to those without antiplatelet therapy (66.7% vs 57.1%, p = 0.353). As demonstrated by Fig 1, this holds true also for all subgroups like BARC3 bleedings (43.8% vs. 33.3%, p = 0.312) or access site bleedings (43.8% vs. 40.5%, p = 0.754). Also after adjustment for confounders (including age, gender, known CAD or PAD, ECMO duration or indication for ECMO) a similar bleeding incidence could be detected.

**Fig 2:**
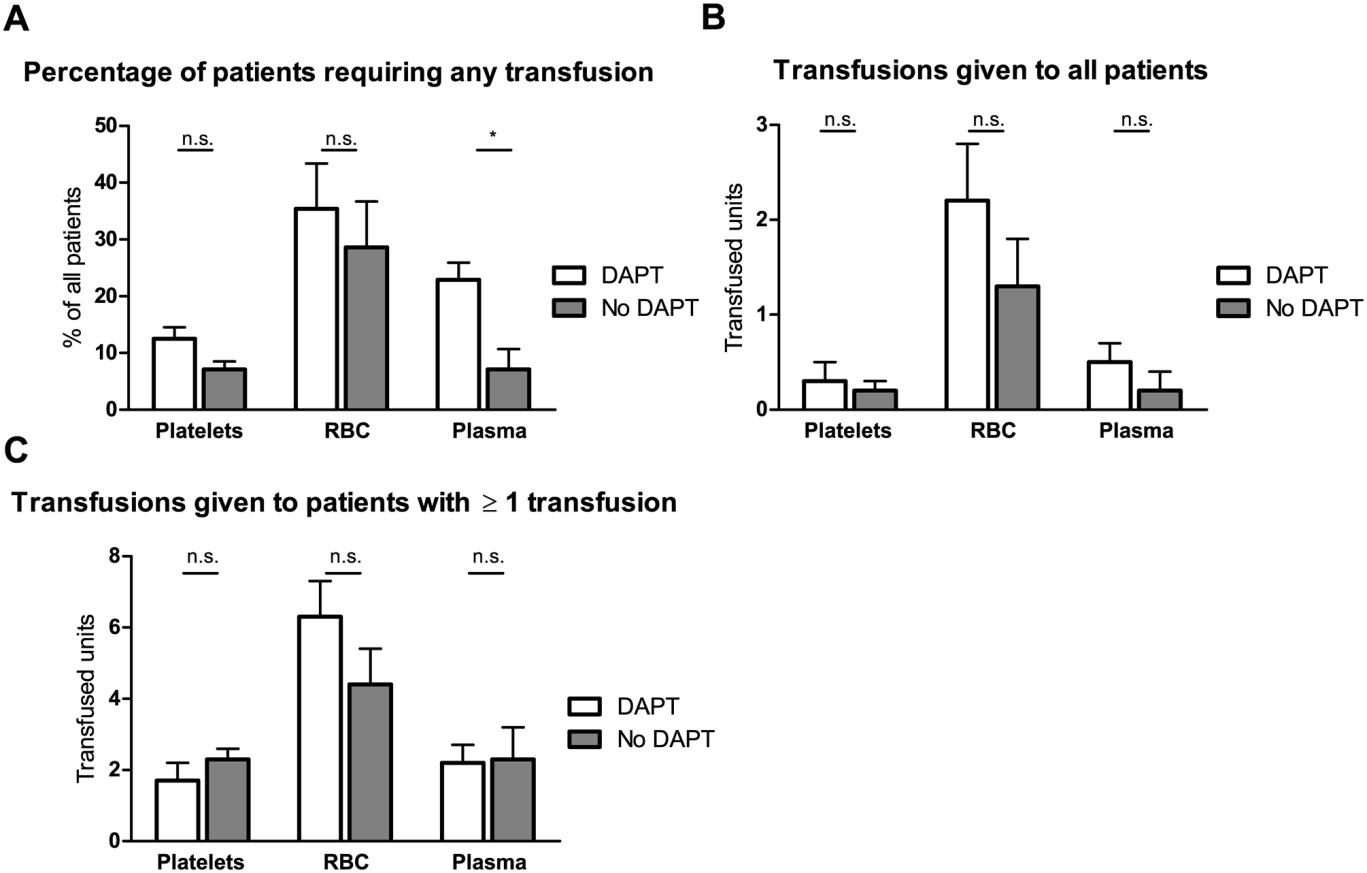
Transfusions of platelets, red blood cells and fresh frozen plasma; Percentage of patients requiring any transfusion (A), Average number of transfused blood products in all patients (B), Average number of transfused blood products in patients receiving at least one transfusion (C).

Transfusion of red blood cells was necessary in 31.2% of patients. The frequency of transfusion did not differ between the groups (35.4% vs. 28.6%, p = 0.488, for DAPT vs no antiplatelet respectively, Table 2 and Fig 2A). Any platelet transfusion was necessary in 9.7% of the patients. Patients in the DAPT group received platelets as often as patients without platelet inhibitors (12.5% vs. 7.1%, p = 0.398). Fresh frozen plasma transfusion was more frequent in the DAPT group (22.9% vs. 7.1%, p = 0.039).

**Table 2:** Bleeding and transfusions.

	All patients	Dual antiplatelet therapy	no antiplatelet therapy	p
Number of patients	93 (100.0%)	48 (51.6%)	42 (45.2%)	
**Bleedings**				
Any bleeding event	56 (60.2%)	32 (66.7%)	24 (57.1%)	0.353
BARC 3 bleeding	34 (36.6%)	21 (43.8%)	14 (33.3%)	0.312
Access site bleeding	38 (40.9%)	21 (43.8%)	17 (40.5%)	0.754
Pulmonary bleeding	16 (17.2%)	8 (16.7%)	8 (19.0%)	0.768
**Transfusions**				
Any RBC transfusion	29 (31.2%)	17 (35.4%)	12 (28.6%)	0.488
Any platelet transfusion	9 (9.7%)	6 (12.5%)	3 (7.1%)	0.398
Any plasma transfusion	14 (15.1%)	11 (22.9%)	3 (7.1%)	**0.039**
**Outcome**				
va-ECMO duration [hours]	55.6 ± 6.9	41.9 ± 1.8	64.2 ± 1.1	0.105
Died on va-ECMO	60 (64.5%)	29 (60.4%)	28 (66.7%)	0.539
Survived	22 (23.7%)	11 (22.9%)	11 (26.2%)	0.718

Bleeding events, amount of transfusions and outcome; statistical analysis is performed for ‘DAPT’ and ‘no any antiplatelet therapy’.

The average number of transfused RBC did not differ significantly between the groups considering all patients (2.23±0.59 vs. 1.26±0.52, p = 0.178, Fig 2B). In patients requiring transfusion, the average number of transfused packed RBC was higher in patients on DAPT (6.29±1.00 vs. 4.42±0.98, Fig 2C) but did not reach statistical significance. There was no difference in platelet transfusion between the groups. Patients on DAPT received significantly more often fresh frozen plasma (22.9% vs. 7.1%, p = 0.039). The average amount of fresh frozen plasma in case of any transfusion was similar (2.17±0.47 vs. 2.33 vs. 0.88, p = 0.87). All patients receiving fresh frozen plasma also received at least one unit of packed RBC.

A total of 23.7% patients survived the initial hospital stay and could be transferred to a rehabilitation or weaning center. There was no significant difference in hospital survival when comparing patients on DAPT with those without and antiplatelet therapy detectable by our registry (22.9% vs. 26.2%, p = 0.72).

Subgroup analysis of bleeding incidence of different P2Y12 Antagonists is given in S1 Table.

## Discussion

Incidence of major hemoglobin relevant bleedings (BARC3) after VA-ECMO implantation was 36.6% in our registry. This incidence is in line with previous reports, where major bleeding on ECMO ranges from 30% [13, 14] to 40% [16, 21] to up to a transfusion of more than 10 packed red blood cells in 54.8% of all ECMO patients [15].

When comparing patients on DAPT to patients without any antiplatelet therapy, we found a slight trend towards an increased bleeding incidence in DAPT patients, which did not reach statistical significance. This holds true for all investigated types of bleedings and after adjustment for all confounders. Complications might have been underreported in this retrospective study. We therefore evaluated transfusions as hard endpoint and as clinical response to bleeding. Transfusion of red blood cells and platelets showed a similar trend towards more transfusion in DAPT patients, again not reaching statistical significance. Significantly more patients on DAPT received fresh frozen plasma transfusions. The average amount of fresh frozen plasma given to each patient however was similar between the groups.

It is an unexpected finding that rate of bleeding and transfusions are similar, since increased bleeding on DAPT has been published for a variety of patient groups not receiving extracorporeal circulation therapy [6, 7, 17, 18, 22, 23]. In venovenous ECMO patients however, the addition of low dose aspirin to unfractionated heparin did not increase bleeding or transfusion [24]. The observed bleeding rates might be partly explained by the fact, that ECMO by itself decreases platelet count and aggregation [11, 12, 25] which mimics platelet inhibition and induces bleeding events by itself [26]. Furthermore, a small increase in bleeding rate might not be detectable in a limited number of patients given the 60% intrinsic bleeding rate in these patients and a medium VA-ECMO duration of less than three days.

The biggest limitation is the retrospective, not randomized character of this study and the limited number of patients. Since there was a significance difference in plasma transfusion and RBC transfusions were numerically more frequent in the DAPT group, RBC transfusions might reach statistical significance in a larger sample size. The two investigated groups represent different patient populations as DAPT was initiated or continued after a significant coronary finding. Randomized data to answer the question if dual antiplatelet therapy leads to a clinically relevant increase in bleeding risk on ECMO therapy will be almost impossible to obtain. The investigated groups were comparable in regard to most patient characteristics including CPR duration and SAPS score. Adjustment for confounders like age, gender, known CAD or PAD, ECMO duration or indication for VA-ECMO did not alter findings.

## Conclusion

Bleeding on VA-ECMO is frequent. A DAPT during short term VA-ECMO had no significant impact on bleeding incidence in this single center registry. On the background of currently published data and our own observations we recommend that DAPT should not be withheld from patients on VA-ECMO due to bleeding concerns. DAPT should be continued or started when indicated by current guidelines even though guidelines do not specifically cover antiplatelet therapy in VA-ECMO patients.

## Supporting Information

S1 File Raw data. Excel file containing raw data.
(XLSX)

S1 Table Impact of different P2Y12 inhibitors. Characteristics and outcome of patients on VA-ECMO by different P2Y12 inhibitors or glycoprotein IIb/IIIa blockage. Data is given as number of patients (percentage of all patients in group) or as mean ± SEM, where applicable. Significance is calculated using ANOVA or Chi^2^ as applicable. CAD—coronary artery disease.
(DOCX)

## Abbreviations

VA-ECMO: venoarterial extracorporeal membrane oxygenation
DAPT: dual antiplatelet therapy
PCI: percutaneous coronary intervention
BARC scale: bleeding academic research consortium scale
INR: international normalization ratio
SAPS2: simplified acute physiology score 2
RBC: red blood cell
