# Supplementary material for: Dual Antiplatelet Therapy (DAPT) versus No Antiplatelet Therapy and Incidence of Major Bleeding in Patients on Venoarterial Extracorporeal Membrane Oxygenation
Source: PLoS One. 2016 Jul 28;11(7):e0159973. doi: 10.1371/journal.pone.0159973 (PMC4965019; doi:10.1371/journal.pone.0159973)
Supplement: S1 Table — Characteristics and outcome of patients on VA-ECMO by different P2Y12 inhibitors or glycoprotein IIb/IIIa blockage. Data is given as number of patients (percentage of all patients in group) or as mean ± SEM, where applicable. Significance is calculated using ANOVA or Chi2 as applicable. CAD—coronary artery disease. (DOCX) [file pone.0159973.s002.docx]

**Supplemental Table 1: Impact of different P2Y12 inhibitors**

|  | Clopidogrel | Ticagrelor | Prasugrel | Eptifibatide | p = |
| --- | --- | --- | --- | --- | --- |
| Number of patients | 15 | 17 | 16 | 13 |  |
| GP IIb/IIIa antagonist | 4 (26.7 %) | 4 (35.3 %) | 5 (31.3 %) | 13 (100 %) | **< 0.001** |
| **Patient characteristics** | | | | | |
| Age [years] | 67.9 ± 3.0 | 67.5 ± 2.1 | 63.1 ± 2.4 | 66.3 ± 2.1 | 0.4706 |
| Male gender | 11 (73.3 %) | 9 (66.7 %) | 12 (80.0 %) | 11 (73.3 %) | 0.2638 |
| SAPS2 Score | 46.2 ± 2.8 | 49.9 ± 2.8 | 51.1 ± 2.9 | 54.7 ± 3.2 | 0.2775 |
| Known CAD | 6 (40.0 %) | 5 (29.4 %) | 6 (43.8 %) | 6 (46.2 %) | 0.8190 |
| Arterial hypertension | 8 (53.3 %) | 8 (47.1 %) | 11 (75.0 %) | 9 (69.2 %) | 0.4950 |
| Kidney disease | 5 (33.3 %) | 4 (29.4 %) | 6 (37.5 %) | 6 (46.2 %) | 0.6234 |
| Diabetes | 4 (26.7 %) | 6 (35.3 %) | 6 (43.8 %) | 6 (46.2 %) | 0.7609 |
| **Complications** | | | | | |
| Any bleeding event | 12 (80.0 %) | 11 (70.6 %) | 8 (50.0 %) | 6 (46.2 %) | 0.2236 |
| BARC 3 bleeding | 10 (66.7 %) | 4 (35.3 %) | 5 (37.5 %) | 4 (30.8 %) | **0.0612** |
| Access site bleeding | 5 (33.3 %) | 6 (41.2 %) | 8 (56.3 %) | 2 (15.4 %) | 0.2815 |
| Pulmonary bleeding | 4 (26.7 %) | 3 (17.6 %) | 1 (6.3 %) | 2 (15.4 %) | 0.4964 |
| Any platelet transfusion | 3 (20.0 %) | 3 (17.6 %) | 0 (0.0 %) | 2 (15.4 %) | 0.3334 |
| Any RBC transfusion | 7 (46.7 %) | 5 (29.4 %) | 5 (31.3 %) | 4 (30.8 %) | 0.7212 |
| Any plasma transfusion | 3 (20.0 %) | 5 (29.4 %) | 3 (18.8 %) | 4 (30.8 %) | 0.8144 |
| **Outcome** | | | | | |
| va-ECMO duration [hours] | 61.2 ± 11.0 | 69.9 ± 17.2 | 60.9 ± 12.3 | 80.9 ± 17.3 | 0.7720 |
| Survived | 5 (33.3 %) | 3 (17.6 %) | 2 (18.8 %) | 0 (0.0 %) | 0.1173 |

Characteristics and outcome of patients on VA-ECMO by different P2Y12 inhibitors or glycoprotein IIb/IIIa blockage. Data is given as number of patients (percentage of all patients in group) or as mean ± SEM, where applicable. Significance is calculated using ANOVA or Chi² as applicable. CAD - coronary artery disease
